# Microbiota and Mucosal Immunity in Amphibians

**DOI:** 10.3389/fimmu.2015.00111

**Published:** 2015-03-13

**Authors:** Bruno M. Colombo, Thibault Scalvenzi, Sarah Benlamara, Nicolas Pollet

**Affiliations:** ^1^Institute of Systems and Synthetic Biology, Université d’Evry Val d’Essonne, Evry, France; ^2^Institute of Systems and Synthetic Biology, CNRS, Evry, France; ^3^Evolution, Genome, Comportement et Ecologie, CNRS, Université Paris-Sud, IRD, Gif-sur-Yvette, France

**Keywords:** small-animal model, *Xenopus*, microbiome, mucosal immunity, chytrid

## Abstract

We know that animals live in a world dominated by bacteria. In the last 20 years, we have learned that microbes are essential regulators of mucosal immunity. Bacteria, archeas, and viruses influence different aspects of mucosal development and function. Yet, the literature mainly covers findings obtained in mammals. In this review, we focus on two major themes that emerge from the comparative analysis of mammals and amphibians. These themes concern: (i) the structure and functions of lymphoid organs and immune cells in amphibians, with a focus on the gut mucosal immune system; and (ii) the characteristics of the amphibian microbiota and its influence on mucosal immunity. Lastly, we propose to use *Xenopus* tadpoles as an alternative small-animal model to improve the fundamental knowledge on immunological functions of gut microbiota.

## The Amphibian Immune System

Amphibians and biologists have a long history in common (1). We learned over the years that amphibians share with mammals the same kind of molecular and cellular immunological mechanisms including components of the innate and adaptive branches of the immune responses (Table 1). The amphibians studied in laboratories are the frogs and toads (*anura*) as well as the salamanders (*urodela*). We know very little on caecilians, the limbless and tailless amphibians. The frogs commonly used in research include *Xenopus*, *Rana*, *Bufo*, and *Hyla* species. The salamanders commonly found in research laboratories are the axolotl (*Ambystoma mexicanum*) and the newt (*Notophthalmus viridescens*). The African clawed frogs *Xenopus laevis* and *X. tropicalis* are by far the most intensively studied amphibians today. Studies on *X. laevis* demonstrated that the amphibian immune system, at least in adult individuals, fundamentally resembles that of mammals (2–4). Little is known about the *X. tropicalis* immune system, however the information obtained from its genome sequence shows that most features of its immune system are similar to those of *X. laevis* (5).

**Table 1:** **Components of the immune system**.

Components	Mammals	Amphibians
Soluble innate immune factors (complement, cytokines)	+	+
Antibody classes	IgA, IgD, IgE, IgG, IgM	IgA/X, IgD, IgF, IgM, IgY
Cells of the innate immune system (Gr^a^, MPh, DC, NK)	+	+
Cells of the adaptive immune system (B and T cells)	+	+
**Unconventional T cells**		
iNKT	+	+^b^
MAIT	+	+^b^
ILCs	+	Few cells (NK); other have not been determined
**Primary lymphoid organs**		
Thymus	+	+
Bone marrow	+	+^c^
**Secondary lymphoid organs**		
Spleen	+	+^d^
Lymph nodes	+	–
Peyer’s patches	+	–
GALT	+	+

*^a^BL, B lymphocytes; DC, dendritic cells; Gr, granulocytes; ILC, innate lymphoid cell; iNKT, invariant NK T cell; MAIT, mucosal-associated invariant T cell; MPh, macrophages; NK, natural killer cells; TL, T lymphocytes*.

*^b^Identification of T-cell subsets with characteristics similar to mammalian counterparts*.

*^c^Absent in tadpoles*.

*^d^In *Xenopus*, spleen is both a primary and secondary lymphoid organ*.

Fertilized amphibian eggs develop outside the mother in the water where they are exposed to microbes during development. Thus, after hatching, the immune system of amphibian embryos needs to develop rapidly. After embryonic development, the amphibian larvae starts metamorphosis and, during this period, the immune system of tadpoles is remodeled (3, 6). The amphibian immune system should enable the replacement of larval-type cells by the adult-type in essentially all the tissues but not at the same time during metamorphosis. At this stage, tadpoles undergo several anatomical and physiological changes, including an increase in glucocorticoids and a significant reduction in the numbers of thymic and splenic T lymphocytes. However, despite the drastic remodeling during metamorphosis, the immunological memory persists through metamorphosis.

### The lymphoid organs

The two primary lymphoid organs found in mammals, thymus and bone marrow, are also present in adult amphibians (Table 2). The source of T lymphocytes in larval and adult amphibians is the thymus. However, B-cell differentiation arises into the liver, while bone marrow, which is more rudimentary than in mammals, essentially supports neutrophil differentiation and contains macrophages precursors (7).

**Table 2:** **Functions of the primary and secondary lymphoid organs**.

Components	Mammals	Amphibians
**Primary lymphoid organs**		
Thymus	T-cell differentiation	T-cell differentiation
Bone marrow	B-cell differentiation	Neutrophil differentiation and presence of macrophage precursors
**Secondary lymphoid organs**		
Spleen	T- and B-cell activation	T- and B-cell activation
		B-cell differentiation in tadpole and adult (in tadpole, B cells differentiate also in liver)
Lymph nodes	T- and B-cell activation	Unknown
GALT	T- and B-cell activation	T- and B-cell activation (unclear)
MLNs	T- and B-cell activation	(Absent structures)
Peyer’s patches	Mostly B-cell activation	(Absent structures)

#### The thymus

In *Xenopus*, the thymus is morphologically recognizable in tadpoles at stage 47, 5 days post-fertilization (dpf) according to the Nieuwkoop and Faber table of development (8). At this stage, the thymus rudiment is made of two spherical masses on each side of the hindbrain and it is colonized by lymphoid precursors. As early as stage 48 (7.5 dpf), the cortex and medulla architecture can be identified. They differentially express major histocompatibility complex class II (MHC-II) molecules on thymic epithelial cells (TECs) and cortical thymocytes express on their surface the cortical thymocyte-specific antigen of *Xenopus* (CTX) (9).

Thymus development is completed by stage 51 (±17 dpf) with many small lymphocytes occupying the cortex and Hassall’s corpuscles, representing groups of medullary TECs (putative mTECs). These structures develop at an earlier stage in the thymus of *X. laevis* than in that of *Rana sylvatica*, and they first appear during metamorphosis (stages 54–66, approximately from 26 to 60 dpf) (10). Hassall’s corpuscles have been proposed, in humans, to act in the removal of apoptotic thymocytes, in the maturation of differentiating thymocytes within the thymus and in regulatory T cell (Treg) induction (11).

Regulatory T cells are involved in the maintenance of the central tolerance, which depends on the promiscuous expression of tissue-specific antigens by terminally differentiated mTECs (12). In humans, mTECs express the transcription factor autoimmune regulator (AIRE) (13), which plays an important role for the central tolerance since its deficiency can lead to autoimmunity. The conservation of the *AIRE* gene has been recently characterized in *X. laevis* and *X. tropicalis* (14).

By stage 55 (±32 dpf), the thymus rudiments are colonized by neural crest-derived pigment cells and shift into a more superficial position, underneath the skin (15). During metamorphosis, the thymus involutes, losing up to 90% of T cells, translocates toward the tympanum and a new wave of stem cell immigration and a second phase of histogenesis occur. Later on in adult life, the thymus can involute as a consequence of aging, estivation, hibernation, or under certain circumstances such as an acute stress (16, 17).

#### Secondary lymphoid organs

In mammals, spleen and lymph nodes are considered secondary lymphoid organs. These organs are involved in the activation of the immune responses. Spleen is also present in amphibians (Table 2). In adult *Xenopus*, the spleen is the main site of B-cell differentiation, while in tadpoles, the liver and the spleen are sites of lymphopoiesis (18, 19) (Table 2). Thus, the spleen is both a primary and secondary lymphoid organ in adult frogs. Moreover, the spleen represents the main peripheral lymphoid organ in amphibians where B and T cells accumulate inside the white pulp.

In *Xenopus*, the spleen appears at stage 49 at about 12 dpf. The mature adult spleen contains regions of red (hematopoietic) and white (lymphopoietic) pulp. During metamorphosis, the spleen cell number reaches a plateau and may even drop even if afterward the organ grows steadily. The spleen is a source of antibody-forming cells. Most of the splenic B cells produce IgM and a very few produce IgY or IgX (2).

Yet, B and T cells, as well as other leukocytes, are found in the liver, the kidneys, and the gut. In *X. laevis* larvae, the liver, the mesonephros, and the ventral cavity bodies contain lymphocytes (20–22). Immature hematopoietic tissue is seen for the first time in mesonephros at stage 48 and lymphomyeloid tissue is seen in the liver at stage 49. Ventral cavity bodies are localized in the anterior part of the tadpole and they occupy the central part of the pharynx by constituting three pairs of lymphoid accumulations in the ventral pharyngeal region (20, 23). The anlagen of the ventral cavity bodies can be distinguished at stage 49 by the presence of hypertrophic cells of the pharyngeal epithelium. The ventral cavity bodies reach their maximum size around stage 56 (±38 dpf) and during metamorphosis they disappear when the branchial apparatus is lost. These structures are possible candidates for the role of bird’s bursa of Fabricius “equivalents.” Yet, they are unlikely central lymphoid organs because of their relatively late appearance, the way in which lymphoid transformation occurs and their thymic dependence. By stage 51, the lymphoid organs of *X. laevis* have completed their lymphoid histogenesis.

In comparison to other amphibians (e.g., *R. catesbeiana*), *X. laevis* larvae seems to lack larval lymph glands. Yet, Mescher et al. described cellular masses connected to the lymphatic system in *Xenopus* tadpoles that may correspond to larval lymph glands (22). But these cellular masses are not well organized and look more like tertiary lymphoid structures than lymph glands. Future studies involving more individuals would be needed to determine if these structures are mainly present when an immune response is ongoing.

Lymph nodes are absent in adult *Xenopus* while they have been described in other anurans (2, 3). Yet, there is a histological description of secondary lymphatic organs in adult *X. laevis* (21). These anatomical structures have been described as diffuse lymphoid tissues in the lamina propria of the gastrointestinal and respiratory tracts as well as in the liver. But they lack clear structural organization of a lymph node with afferent and efferent lymphatic vessels. Thus, they could rather be described as tertiary lymphoid organs or diffuse lymphoid tissues, as they can be sometimes observed in the kidneys and lungs. Still neither Peyer’s patches (PPs) nor mesenteric lymph nodes (MLNs) were described in the intestine, although dispersed lymphoid aggregates were observed throughout the intestinal epithelium (24, 25).

Isolated lymphoid follicles (ILFs) have been found in all vertebrates, including amphibians, reptiles, and birds (24, 26, 27). These structures have been suggested to be equivalent to the intestinal induced lymphoid tissues found in the small intestine of the mammals (28, 29). In mammalian fetuses, the development of secondary lymphoid organs is programed, but the formation of other lymphoid structures, such as ILFs in the gut or tertiary lymphoid tissues can be induced after birth by external signals (30). These tissues have been associated to pathological processes in mammals: autoimmunity, chronic inflammation, infections, and cancer. Yet, these tissues can also contribute to the local protective immune response (31). It is possible that the pathways used to form ILFs in non-mammalian vertebrates serves in mammals for the development of lymph nodes and PPs, as previously suggested (32). Nevertheless, in non-mammalian vertebrates, notably *Xenopus*, studies on the cytokine signaling involved in the differentiation of these structures have not yet been undertaken. However, this evolutionary conservation could explain the capacity of non-mammalian vertebrates to discriminate between the commensal and pathogenic gut microbiota.

### The innate and adaptive immunity

#### Innate immunity

Innate immunity emerged early during evolution and represents the first line of defense of the organism against pathogens (33). This branch of the immune system involves different cell types and many soluble factors (e.g., INO, ROs, etc.) and cell surface or intracellular receptors (e.g., Fc-receptors, TLRs, NOD, etc.). As for mammals, the effector cells of amphibian innate immunity eliminate infected cells by phagocytosis, *via* macrophages, neutrophils, and dendritic cells (DCs) or by natural killer (NK)-mediated direct cytotoxicity. Moreover, the humoral side of the innate immunity in amphibian includes epithelia-secreted antimicrobial peptides and some serum peptides, including those of the complement system (3). The abundance and activity of several epithelial peptides suggest that antimicrobial peptides play a key role in host defense of all mucosal surfaces even though the literature is mainly focused on skin mucosa (34–37). The mucus layer is also an important component to protect epithelia from infections. The mucus in the small intestine contains high concentrations of antibacterial peptides, such as defensins and lysozymes, and complements a lack of physical barriers at the level of the crypts. These proteins are secreted by both Paneth cells and enterocytes and generate a gradient of antibacterial substances from the epithelial side (38, 39). Similarly to mammals, amphibians produce high levels of lysozymes with antimicrobial activity. Several lysozyme genes are expressed in several tissues including the skin and the egg (40, 41).

#### Adaptive immunity

Innate immunity in all vertebrates also plays a critical role in the initiation of the adaptive immune response, which is specific of a given foreign antigen. Adaptive immunity represents the most recent branch of the immune response from an evolutionary point of view, since it appeared in gnathostomes (42). B and T lymphocytes are the cells belonging to the adaptive immune system. As for mammals, T cells from *Xenopus* are divided in helper CD4^+^ and cytotoxic CD8^+^ cells. Both B and T cells possess on their surfaces antigen-specific receptors (BCR and TCR, respectively), which are able to recognize an array of different antigens. Activated B cells secrete antibodies, which represent the humoral side of the adaptive immunity able to neutralize foreign antigens.

The extended studies on both innate and adaptive immunity in *X. laevis* provided the basis for the analysis of *X. tropicalis* genomic sequence. Many genes involved in mammalian innate or adaptive immunity have been identified in amphibians, including both *X. laevis* and *X. tropicalis* (3).

### T and B lymphocyte differentiation

#### T cells differentiation

A thymocyte differentiation pathway has been characterized by using a panel of *X. laevis*-specific mAbs recognizing CD8, CD5, and CD45, in association with a *X. laevis* mAb recognizing the CTX molecule as a surface marker of immature thymocytes (9, 43). As in mammals, *Xenopus* thymic ontogeny is characterized by successive waves of thymocytes moving into the thymus where they expand, differentiate into mature naive T cells, which are then exported in the periphery. The first steps identified in *X. laevis* concerns the differentiation of immature DP-like (CTX^+^, CD8^+^, CD5^low^, CD45^low^) into more mature SP-like stage (CTX^−^, CD5^bright^, CD45^bright^) that could be further subdivided into CD8^bright^ and CD8^−^ (i.e., CD4^+^) T cells (44). CD8b and CD4 gene expression can be detected at the time of thymic organogenesis, thus indicating that CD8 and CD4 T-cell differentiation takes place in tadpoles (45).

As indicated above (see The Thymus), tadpoles undergo a significant reduction of thymic and splenic T cells during metamorphosis. A second wave of stem cell immigration occurs just after metamorphic completion (stage 66, ±58 dpf) (46, 47). New thymocyte precursors differentiate in the thymus in young post-metamorphic adults (43–45, 48). As this new intrathymic differentiation arises in a different environment, a new “adult-type” education including negative selection by the adult self is given to the emerging adult T cells. Thus, the adult organism obtains a new balance of self-tolerance.

Major histocompatibility complex class-I and -II molecules are differentially expressed between tadpoles and adults. Classical MHC-Ia molecules are not detectable by antibodies in tadpole thymus before metamorphosis and their expression is firstly detectable on erythrocytes and splenocytes at metamorphic stages (49, 50). Nevertheless, some class Ia mRNA are detected in tadpole thymus (51). MHC-II expression was reported on B cells and leukocytes located in the thymic medulla of both tadpoles and adults. Interestingly, no expression of MHC-II has been detected in tadpole thymocytes and T cells, whereas adult T cells are positive for MHC-II (52). Experimental impairment of MHC class Ia expression in mammals would lead to immunodeficiency or death. Yet, tadpoles develop normally in spite of a deficient or suboptimal class Ia-restricted thymic education. Moreover, *Xenopus* pre-metamorphic tadpoles are immunocompetent and have circulating CD8^+^ T cells. The mechanism that may explain tadpole survival with suboptimal MHC class Ia expression could be the use of non-classical MHC molecules in tadpole thymic T-cell education and an overall more limited TCR repertoire in tadpoles (43).

#### B cells differentiation

B-cell differentiation occurs early in the development of amphibians in comparison to mammals. As in mammals, three differentiation stages of *Xenopus* B cells can be found: pre-immune B cells that express only IgM heavy chain, B cells expressing heavy and light chain, and terminally differentiated plasmocytes that are surface Ig negative and secrete immunoglobulins. Two main periods characterize amphibian B-cell ontogeny. The first period leads to a pre-immune B repertoire composed of less than 100 pre-immune B cells. This first period starts in the liver at the larval stage, at approximately 5 days after fertilization (stage 47). The pre-immune B cells are able to recognize several antigens and each clone is different, considering the low number of cells and the diversity of recombination segments (53). During this period, IgM heavy chain recombination depends exclusively on intrinsic signals. After this period, a limited number of pre-immune B cells (because there are not many progenitors) are positively selected for a productive recombination of IgM heavy chain, and will constitute the pre-immune B repertoire in the liver. The second period of B cells ontogeny starts 12 days after fertilization (stage 49) with the spleen differentiation. This period leads to the acquisition of immunocompetence, and will continue even after metamorphosis.

The first mature B cells are detected approximately 10 days after fertilization (stages 48–49) with the onset of light chain recombination. After 2 weeks of development (stage 49–50), B cells expressing a complete IgM can be found mainly in *Xenopus* liver and spleen. Nevertheless, no B cells are found in the gut mucosa at larval stage, whereas B cells are present in the adult gut, as well as in adult thymus and bone marrow. The bone marrow is not hematopoietic during ontogeny in amphibians since tadpoles lack bones and bone marrow. Furthermore, tadpoles’ plasma cells do not reach the same level of differentiation as adult ones (54).

The structure of amphibians and mammalians Igs is similar, with constant and variable domains for heavy (CH and VH) and light (CL and VL) chains. The same mechanisms of VDJ segment recombination occur in *Xenopus* and in mammals for Igs formation. Similarly, heavy chain genes recombine before light chain genes. There are a hundred of VH genes corresponding to 11 families, more than 10 DH genes and 8–9 JH genes. The exact number varies depending on the considered species: for example, only five DH segments and seven JH segment have been identified so far in *X. tropicalis* (55). The recombination profile of Igs differs between tadpoles and adults. At larval stage, VH1 family is preferentially expressed. This resembles the expression of the mammalian VH3 ortholog during early ontogeny. The combination of DH1 and JH3 segments is also observed as there is no N nucleotide insertion at larval stage, so homology-based V(D)J junctions seem to be more frequent.

Knowing the *X. tropicalis* genome sequence led to a better understanding of Ig genes localization and relative order. Bioinformatic studies revealed that heavy chains of Ig genes are found on two nearby chromosomic loci (scaffold_972 containing the constant region of IgF, and scaffold_928 containing the constant regions of IgM, X, Y, and D). A reconstitution of gene order of the IgH locus has been made, suggesting a similar organization of mammalian and *Xenopus* genes (cluster of VH–DH–JH–CH genes) (55).

As in mammals, an isotype class switch is observed in *X. laevis* but it is not found in *urodeles* (56). In comparison to the five Ig isotypes known in mammals, three isotypes were first described in *Xenopus*: IgM, IgY, and IgX. *Xenopus* IgM, the most abundant isotype, is analogous to the mammalian IgM and the IgY isotype is analogous to mammalian IgG. However, more recently, *X. tropicalis* genome sequence allowed the identification of two additional Ig isotypes: IgD and IgF. Bioinformatic and phylogenetic studies has showed that *Xenopus* IgD was the homolog of mammalian and fish IgD. The isotype IgF is not analog to any mammalian Ig type. Molecular studies reveal that IgD and IgF are mainly found in *Xenopus* spleen. Interestingly, sequences corresponding to Ig hinge regions were identified in *X. tropicalis*, with the discovery of IgF. Hinge regions were known in mammals, and identified in the teleost fish *Fugu*. These results bring novel data to study the evolution of immunoglobulin functions (55).

As we are interested in mucosal adaptive immune system, we will focus on the mucosal Ig isotype identified in *Xenopus* as IgX. Phylogenetic studies of immunoglobulin heavy chain constant region showed that IgX is closer to mammalian IgA than mammalian IgM (57). Thus, IgX is considered the ortholog of the mammalian mucosal IgA isotype. Nevertheless, IgX and IgA appear to be structurally different, as IgX is composed of four heavy chain constant domains, whereas mammalian IgA has only three (55). Functional studies show an increase of serum IgX after oral immunization with cholera toxin whereas no significant changes are observed after intra-celomic injection. These results suggest an induction of mucosal immune responses against mucosal pathogens through IgX in *X. laevis*. Moreover, IgX is preferentially expressed in the frog gut, since IgX B cells were found in a more abundant proportion in this tissue compared to other tissue such as spleen and liver. IgM B cells have also been described in *Xenopus* gut epithelium. However, this tissue does not seem to contain any IgY B cells (58). Interestingly, IgX levels in lymphocyte cultures derived from the intestine does not seem to be affected by larval thymectomy, suggesting the existence of T-independent mucosal immune responses in the frog gut (57). IgX, but also IgM and IgY are found in *Xenopus* skin mucus after immunization with a chytrid fungal pathogen of amphibian skin (59). These data suggest the implication of Ig in skin immune responses, as shown in mammals and in fish.

### The gut mucosal immune system

#### Anatomy of the alimentary tract

The alimentary tract of a 5-days old *Xenopus* tadpole contains a pharynx, esophagus, stomach, intestines, pancreas, and liver and therefore it is similar to its mammalian counterpart. The ontogeny of these organs has been described in great details for *Xenopus* (60–62). A single layer of primary epithelium forms the intestine of tadpoles. This epithelium is organized in a simple tubular structure, with a central fold called the typhlosole. Upon metamorphosis, the tadpole’s intestine changes into a more complex adult intestine (63). This adult intestine is made of a multi-folded epithelium surrounded by connective tissue and muscles. Morphologically, the frog small intestine contains a network of mucosal folds running throughout its length. In this epithelium, four cell types resting on a continuous basement lamina have been initially described: columnar cells, goblet cells, endocrine cells, and leukocytes (64). Cells located in the pits and crest cells of the intestinal folds exhibit differences in functional activity (65). Thus, the amphibian adult intestine resembles adult mammalian intestine, and functions in a similar way. In addition to the four cell types described initially, 1 mm large granular glands formed during metamorphosis were found in the epithelium of the gastrointestinal tract. These granular glands are syncytium made of large multinucleated, granule-filled cell containing a variety of biologically active peptides, including antimicrobial peptides (65, 66). In addition, cells resembling mammalian Paneth cells have been found in the frog intestine (66). As in mammals, the goblet cells of the gut secrete a protective mucus layer into the lumen to prevent infection and support the normal microflora. As indicated above, amphibians do not present PPs and MLN but intraepithelial lymphocytes and ILFs in tadpoles or adults (24, 25) (Table 3).

**Table 3:** **Composition of the mucosa-associated lymphoid tissues (MALTs)**.

Components of the MALTs	Mammals	Amphibians
**Scattered lymphoid cells**		
IEL^a^	+	+
**Organized lymphoid tissues**		
M cells	+	–
Peyer’s patches	+	–
MLNs	+	–
ILF	+	+
Secretory Ig	IgA	IgX (functional analog of IgA)

*^a^IEL, intraepithelial lymphocytes; Ig, immunoglobulins; ILF, isolated lymphoid follicle*.

#### Background on the gut mucosal immune system

The gut mucosal immune system forms the largest vertebrate immune compartment (67). It is now well established that its functions depend partly on the presence of intestinal microbes, constituting the so-called microbiota or intestinal flora. The intestinal microbiota is mainly composed of bacteria, which can represent a natural defense barrier exerting different protective, structural, and metabolic effects on the host epithelium (68, 69). Intestinal bacteria benefit from a stable environment and the host gains digestive and metabolic capabilities. This symbiosis establishes an “immunological paradox” forcing the host to combine tolerance to commensal microbiota *via* regulatory-suppressive immune response and rapid recognition and fight of pathogens *via* effector mechanisms (70).

We still do not know how the host immune system distinguishes when a given microbe becomes pathogenic. We know that macrophages and DCs can recognize and discriminate between microbial-associated molecular patterns (MAMPs such as LPS, flagellin) or damage-associated molecular patterns (DAMPs, such as stress or injury) *via* specialized membrane and cytoplasmic pattern recognition receptors (PRR, such as TLRs and NODs) (71). Thus, components of the innate immunity can drive a response toward anti-inflammatory or pro-inflammatory mediators, which can then induce tolerance or inflammation, respectively.

The capacity of the adaptive immunity to recognize and respond to specific microorganisms seems to be driven by the microbiota itself. This response leads to the clearance of the pathogens and helps microbiota itself to improve host health. Thus, gut bacteria profoundly influence immunologic well being.

**Figure 1:**
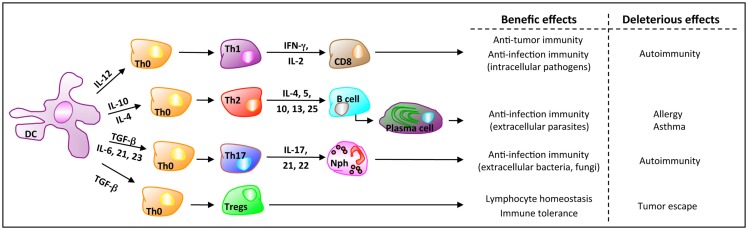
**Orchestration of the mucosal immune system**. Based on the environmental signals, DCs secrete cytokines that are critical for the gut immunity. Here are represented the four main CD4^+^ T-cell populations orchestrated by DCs and the cytokines involved in their differentiation, for host defense against danger signals (Th1, Th2, Th17, which are effector T cells) and for the regulation of the homeostasis (Tregs). Once homeostatic control mechanisms are impaired, effector T cells activity can be deleterious. Furthermore, based on their suppression activity against autoreactive T cells, Tregs may favor cancer progression because of the suppression of anti-tumor (autoreactive) T cells.

In mammals, balanced mucosal immunity in the gut is critical for host homeostasis and defense. This balance mainly depends on three cell populations: DCs, effector T cells (either CD8^+^ or conventional CD4^+^ lymphocytes), and Tregs (Figure 1). At the steady state, i.e., without any danger signal, DCs are tolerogenic. In MLNs, they promote the differentiation of naive CD4^+^ T cells toward Tregs. Intestinal tolerogenic DCs express the integrin molecule CD103 and have an enhanced capacity to metabolize the dietary vitamin A into retinoic acid (RA). RA is a pivotal signaling molecule involved in mitigating inflammation by inducing Tregs activity (72). Pro-inflammatory Th cells play a crucial role in clearing pathogens during host defense reactions but can also induce tissue inflammation and subsequent tissue destruction, notably in an autoimmune context. Tregs are recognized to be one of the major regulatory element or player in immune tolerance and inflammation. Accordingly, the imbalance and dysregulation of Tregs and pro-inflammatory Th cells in the intestine is closely associated with intestinal autoimmune disorders like the inflammatory bowel disease (IBD). At the same time, it is now recognized that the microbiota regulates both T-cell subsets in rodents and, possibly, in humans (73).

Although helper T-cell functions have been characterized in *X. laevis* adults, little is still known about CD4^+^ T-cell subsets. This is due to the lack of a CD4 antibody for use in amphibians. But a *CD4* ortholog is present in the *Xenopus* genome, and CD4 transcripts have been identified (45). Treg cells have been reported on *X. laevis* by detecting the presence of a *foxp3* transcript and in *X. tropicalis* by detecting the presence of a gene ortholog with a well-conserved genetic synteny (74–76). Moreover, the *Xenopus* genome contains an ortholog of the interleukin 2 receptor alpha gene, equivalent to CD25. Treg function during metamorphosis in *Xenopus* tadpoles is suggested by the tolerance to grafts of adult skin with minor histocompatibility antigens. These grafts are not rejected and remain in adults. This tolerance is specific since a third graft with a genetically different donor is rejected. Together, these results indicate an active tolerance mechanism since it can be abrogated by treatment with cyclophosphamide and can also be adoptively transferred (3). Despite the conservation of immune cells and their functions in gnathostomes, very little is known about the mucosal immunity balance between effector T cells and Tregs. Nevertheless, a recent study demonstrated that *X. laevis* metamorphosis is an excellent model system for studying mammalian gastrointestinal development. Indeed, this model was used to identify the genes and signaling pathways that are essential for intestinal development and maturation, including genes associated with the immune responses (63).

T lymphocyte subsets bearing TCRs with invariant alpha-chains are also evolutionary conserved and are thus indicative of specialized functions (Table 1). These class Ib-restricted T-cell subsets include the CD1d-restricted invariant NKT (iNKT) and the MHC class I-related molecule-restricted mucosal-associated invariant T (MAIT) cells. These unconventional T cells respond to a wide variety of different microbes (bacteria, parasites, viruses, and fungi) and have antimicrobial activity, suggesting a role of these cells during microbial infections (77). More particularly, MAIT cells reside primarily in the gut lamina propria and require commensal flora for selection/expansion (78). T-cell subsets with characteristics similar to mammalian iNKT and MAIT cells have been recently described also in *Xenopus* and have specialized functions early in immune responses, in unmanipulated frogs and tadpoles (79).

Innate lymphoid cells (ILCs) are also involved in the control of the intestinal homeostasis in mammals (80). They have been divided into three groups, based on their ability to produce type 1, type 2, and Th17 cell-associated cytokines (81). NK cells, belonging to the group 1 ILCs, have been identified also in *Xenopus* (82) (Table 1). Nevertheless, both group 2 and 3 ILCs were described only in mammals. Importantly, group 3 ILCs plays a crucial role in mediating the balance between microbiota and the intestinal immune system. This group comprises a polyfunctional cell population, the lymphoid-tissue inducer (LTi) cells, which is also involved in the formation of secondary lymphoid organs during embryogenesis. Depletion of group 3 ILCs results in peripheral dissemination of commensal bacteria and systemic inflammation (83).

Beside DCs, T cells, MAIT cells, and ILCs, non-mammalian vertebrates also share B cells and antimicrobial peptides with mammals. The adult gut mucosal immune system is thus composed by plasma cells producing secretory IgA in birds and mammals, IgT in teleost fish, and IgX in amphibians (84). B cells in *Xenopus* express three immunoglobulin isotypes (IgM, IgX, IgY). Large numbers of IgM- and IgX-, but not IgY-, positive B cells are located in the gut epithelium of the intestine. In this organ, up to 60% of all B cells can be IgX positive and the majority of IgX-producing cells morphologically resemble plasma cells. They are secretory B cells, but they do not lose membrane Ig expression (57, 58). Finally, amphibians have the ability to produce antimicrobial peptides, as mentioned previously.

### Skin, ovary, and other mucosal systems

Frogs and salamanders are well appreciated because their skin is covered by an abundant mucous produced by numerous mucous glands. Both mucous glands and the less abundant granular glands are located over the entire body. Secretions from these glands protect the skin from mechanic trauma, inhibits entry of pathogens, and play osmoregulatory roles. Different mucous layers cover tadpole and adult skins. The adult skin consists of an epidermis and a dermis. The epidermis is a keratinized stratified epithelium. The dermis is considerably thinner than in mammals and is made of a loose connective tissue layer underneath the epidermis and of a dense connective tissue. This dense connective tissue contains exocrine glands, including mucous glands and the same granular glands found in the gastrointestinal tract. The skin mucous chiefly serves as a protective layer but also facilitates a proper salt and water balance within the internal organs when the amphibian is in water (85). Granular glands secrete a variety of substances, including toxins, pheromones, and antimicrobial substances. Toxins such as neurotoxins, cardiotoxins, and hallucinogens play a role in the defense against predation. Antimicrobial compounds are components of an innate immune response and defend amphibians against bacterial and fungal infections. In addition, amphibians regularly experience skin sloughing and this regulates the population of skin microorganisms (86). At every episode of sloughing, the skin microbiome population becomes reduced and returns to a basic level. Thus, risks of dysbiosis events on the skin are reduced. Since many amphibians eat their skin, it is possible that a complex cycle is established between the skin and the gut microbiome.

In *X. tropicalis*, ciliated and intercalating non-ciliated cells are located in the inner epidermal layer in embryos during the early neurula stage (stage 14, approximately 16 h post-fertilization), while both cell types intercalate into the outer layer by the late neurula stage (stage 25, corresponding to 1 dpf). At stage 27 (1 dpf), ciliated cells and goblet cells are recognizable in embryonic skin, which is constituted by approximately 60% of goblet cells, 18% of ciliated cells, and approximately 22% of intercalating non-ciliated cells (85).

The respiratory epithelium of the nasal cavities and of the trachea is also lined up by a pseudostratified epithelium and underlying lamina propria. Goblet cells are found all over the respiratory epithelium with varying densities. In addition, the olfactory epithelium is covered by a mucous secreted by the Bowman’s gland. The perilymphatic cistern of the inner ear is lined up by the same kind of pseudostratified epithelium as in the nasal cavities. In the oviduct, many glands empty their contents in the lumen as oocytes travel. These secretions contain mucin-like glycoproteins and will form the jelly coat of the oocytes (21).

An experimental challenge in the *Xenopus* amphibian model is the lack of monoclonal antibodies as molecular probes for various immune-related molecules. Nevertheless, some are available through the University of Rochester resource on *Xenopus* immunology and enabled immunohistochemistry studies. Thus, some mucosal cell populations have been identified. A population of cells expressing formalin-resistant ATPase can be observed starting at stage 28 (1 dpf) in *Xenopus* larval skin (22). These cells have been assumed to be equivalent to mammalian DCs and Langerhans cells, depending on their localization in the dermis or in the epidermis, respectively. Cells with similar morphology and localization in *Xenopus* embryonic skin have been found to express vimentin, between stages 28 and 37 (1–2 dpf). Vimentin, a cytoskeleton protein involved in remodeling, is highly expressed in immature DC in mammals. These two observations suggest a tolerogenic potential of the vimentin positive cells, since immature DC are not able to activate T lymphocytes but induce immune tolerance (87). These findings suggest the presence of DCs and Langerhans cells in *Xenopus* skin, but also a maturation process of these cells, as observed in mammals. DCs and Langerhans cells have also been observed in other amphibian species, such as *R. pipiens* (88).

A population of cells expressing the highly conserved epsilon chain of CD3 has been found in *Xenopus* epidermis starting at 5 dpf (stage 47). These cells are more abundant in *Xenopus* epidermis at pro-metamorphic stages, occurring from 2 to 26 dpf (stage 35/36 to stage 54), and in *Xenopus* gut. Interestingly, cells with similar shapes and distributions have been found to express delta TCR chain (22). Because of their phenotype, morphology, and localization, these cells have been assumed to be equivalent to dendritic epidermal T cells (DETCs). DETCs are known to express gamma delta TCR and are mucosal cells implied in early peripheral immune defenses against pathogens. DETCs are also implicated in host tissue maintenance and cell homeostasis in mammals. Indeed, they avoid tissue damage after inflammation by cytokine immunoregulation, and are involved in tumoral immunosurveillance. They also play a crucial role in wound healing (89).

Another interesting discovery is the agglomeration of CD3 positive cells in pectoral and pelvic areas of stage 50 (15 dpf) tadpoles, very close to the limbs. These agglomerates are associated with the epidermis but also with endothelial lymphatic structures. Thus, they remind the lymph glands of *Rana* tadpoles and the mammalian lymph nodes. Nevertheless, no follicular structures were observed and functional studies of these CD3 positive structures await future studies.

Cells expressing MHC-II molecules have been found scattered in stage 54 (26 dpf) *Xenopus* epidermis. MHC-II positive cells have also been found in larval and adult gut epithelium, and spread over the pharyngo-buccal cavity epithelium (52).

## The Amphibian Microbiota and Mucosal Immunity

Microorganisms dominate our external environment and some of them live on and in ourselves, as well as on and in animals. These microorganisms can be symbiotic, sharing a mutual beneficial relationship with the host, commensals, thus neutral, or parasites and opportunistic, causing pathologies. Overall, “healthy” symbionts constitute the so-called commensal microflora. This microflora participate to the architecture and function of the colonized tissues such as skin and mucosa (90). Commensal microbiota include bacteria, archaea, fungi, viruses, protozoans, and, in some cases, multicellular eukaryotes such as helminths. However, bacteria predominate in number and diversity and can reach 100 trillion microbial cells in the mammalian colon. The relationship between microbiota and the mucosal immune system has been extensively studied in humans and several other mammals (91–93).

### The microbiome

The sum of the microbiota genome sequences is called the microbiome. In humans, the microbiome contains 3.10^6^ genes, i.e., more than 150 times as many genes as in the human genome. Our gut microbiota likely contains 1000 to 1150 bacterial species (spread among all people sampled), with each person harboring about 160 bacterial species, thus suggesting that an individual’s microbiome is relatively distinct in composition and is adaptable to environmental changes and/or host genetics (94). While the human microbiome is the subject of intense scrutiny, there remains a need to compare the human microbiome with that of other animals. Such comparisons could lead to the development of *in vivo* models where the host, microbial, and environmental parameters can be controlled (95). For example, some pioneering studies on zebrafish and *Drosophila* demonstrated that microbiota participate to the host metabolism (96, 97). Thus, this comparison would be helpful to identify emerging diseases from natural populations and to explore animal–bacterial interactions (98).

The indigenous flora hosted by amphibians is not well known, in particular there is only few publications reporting on the diversity and the function of microorganisms in tadpole and adult amphibian physiology. What we know so far is derived to a large extant from studies of the skin and to a lesser extant of the gut microbiome.

### Amphibian skin microbiome

Amphibians make extensive contacts with microorganisms from soil and water. Thus, their skin microbiota depends on each species habitat. While studies on natural populations have been undertaken to know which bacteria are members of amphibian skin microbiota, we lack such a knowledge for laboratory-reared amphibian model species including *Xenopus*.

Ironically, our knowledge on the amphibian skin microbiome owes a lot to *Batrachochytrium dendrobatidis*, a dreadful amphibian skin pathogen. This pathogen is a chytrid fungus and it causes the skin disease chytridiomycosis (99–102). Chytridiomycosis has spread all over the planet and is one of the causes that leads to massive amphibian deaths (103). However, some amphibian species exposed to chytrids can develop resistance to the disease. We now know that this resistance is in part due to differences in the composition of the skin microbiome. Pathogenic fungi infections are effectively inhibited by small molecules produced by the cutaneous bacterial flora of amphibians such as the salamanders *Plethodon cinereus* and *Hemidactylium scutatum*. Bacterial isolates present on the salamander skin are able to secrete compounds active against both *B. dendrobatidis* and ascomycete fungi (104). At least 10 genera of bacteria are able to inhibit *B. dendrobatidis* growth *in vitro* and are effective against other fungi.

In a study of the natural bacterial microflora found on the skin of newts, salamanders, and bullfrog tadpoles, the authors isolated only one bacterium, *Pseudomonas fluorescens*, from a completely aquatic species and from completely terrestrial species (105). The other bacteria isolated were unique to each batrachian species and were common in the environment, aquatic or terrestrial, and mostly of the mucoid type. A few opportunistic pathogens commonly part of the normal flora, such as *Aeromonas hydrophila* and *Chryseomonas luteola* were also isolated (106). The authors remarked variations of the skin microbiome according to the body location: ventral or dorsal. These differences reflect the environment in contact with the skin and the heterogeneity of skin gland density on the ventral and dorsal sides of the animals.

While several studies identified bacterial species growing *in vitro* from amphibian skin, more recent work aimed to identify the non-growing species of the skin microbiome. Several studies aimed to investigate the diversity of amphibian skin microbiomes in natural populations (107–109). In the most recent survey, the authors sampled six geographical sites and four different species (two anurans *Lithobates catesbeianus* and two urodeles *Taricha torosa*) from California’s central valley (108). In addition, they sampled 12 geographical sites from northern California where they collected different developmental stages of the frog *R. cascadae*. The authors sequenced 16S PCR products obtained either from skin swabs or from water, sediment, and soil samples. They found that the skin microbiote composition diverged significantly between amphibian species. The most abundant bacteria were Bacteroidetes, Gammaproteobacteria, Alphaproteobacteria, Firmicutes, Sphingobacteria, and Actinobacteria. In *R. cascadae*, metamorphosis was associated with a shift in the skin microbiome. Tadpole’s skin was rich in the same *Pseudomonas* taxon commonly found in the water they lived in. In adult amphibians, the bacteria from the environment were not a significant factor of skin microbiome diversity. Thus, it seems that there is a vertical transmission of skin microbiome within these species (108). Yet, there is an apparent paradox between the tadpole situation in which the skin microbiome resembles that of water, and the adult one in which the skin microbiome is different from that of the environment.

The microbiome community dynamics on the skin of the salamander *Plethodon cinereus* was further explored (107). The authors compared the bacterial population of the soil to that of the salamander skin in their native environment or in the laboratory. They observed a change of the microbiota composition when the salamanders were brought to the laboratory. This change was observed whether the salamanders were kept with their soil of origin or in a sterile media. This change may reflect the stress of capture and food change, since it is likely associated with the release of skin gland secretions. The authors identified a core microbiome that inhabits the skin of more than 90% of salamanders. This core microbiome is composed of eight taxonomical groups and is also found in the soil. In this core microbiome, the gamma proteobacteria *Pseudomonadaceae* are predominant since they represent five of the eight taxonomical units. The bacterial families *Staphylococcaceae* (commonly found on human skin), *Comamonadaceae*, and *Opitutuae* were also represented in this core. Salamanders raised in an environment without soil developed a skin microbiota rich in *Verrucomicrobia* and less diverse than the core microbiota. Overall, this study’s results question the relevance of analyzing the skin microbiome of amphibians outside of their native habitat. Also, it shows that the soil supports the presence of rare microbial species competing with a core set of commensal bacteria. Once in the laboratory and without a bacterial reservoir, the core microbiome composed 93.5% of the skin microbiome. A few commensal species could then dominate the skin microbiome such as the *Opitutuae*, and this may eventually lead to a dysbiosis. This may be relevant for conservation biology of amphibians.

The knowledge of the skin microbiome in chytrid-tolerant amphibians has enabled the development of probiotic therapies to fight chytridiomycosis (110–113). The failure of some trials lead to take into more considerations the intricate relationships between environmental conditions, microbial communities, immune functions, and probiotic therapies. In an interesting study, Woodhams and colleagues explored the interactions between the skin microbiota, immunity, and the composition of the mucosal secretions, termed the “mucosome” (114). Woodhams and colleagues define the mucosome as the humoral component of the skin mucous, be it derived by the host molecular secretions (antibodies, lysozyme, mucins, peptides, small molecules) or by the microbiota (secondary metabolites). The authors identified amphibian natural populations differing by their infectious status and their sensitivity to chytrid infections. By systematically investigating chytrid infections and the efficiency of the skin immune responses, Woodhams et al. revealed that infections can be correlated with the antifungal activity of peptides released by the granular glands and of skin mucosal extract. In addition, the authors showed that a probiotic therapy could influence the anti-Bd efficiency of the skin mucosome functions. Overall, this study identified several factors to take into account for probiotic therapy development. In particular, the interactions between species-specific factors and environmental conditions make clear that *in vivo* testing of probiotics is an absolute prerequisite for the development of successful therapies.

### Amphibian gut microbiome

In comparison to other animals, we know little on the feeding ecology of tadpoles. The majority of tadpoles found in ponds are suspension feeders. Microorganisms such as algae, bacteria, fungi, phyto and zooplankton, and detritus compose their natural diet. Thus, most tadpoles can be classified as herbivorous or microcarnivorous. Yet, some tadpoles are macrocarnivorous, and others can vary their diet according to available resources (115). In all cases, tadpoles’ gut contains all kinds of microorganisms, mostly bacteria and algae (116–118).

The first reports on the existence of a microbial flora in the amphibian hindgut dates back to the 1960s (119). Later, Pryor and Bjorndal published a seminal paper describing precisely the gut structure, digesta passage, and fermentation in an anuran tadpole with an herbivorous diet. They reported that the bullfrog larval gut is more than 10 times longer than the body length. This gut is voluminous and contains a significant symbiont community. The gut anatomy enables a slow rate of digesta passage and allows more time for symbiotic fermentation. In addition, a mucous matrix covers the gut epithelial surface where most of the microflora is found. This microbial community is active and provides 20% of the tadpole’s daily energy requirement by fermentation. Several small chain fatty acids such as acetate, butyrate, and propionate are produced by fermentation in the small intestine and in the colon (117). The microbiodiversity of the gut in these tadpoles is complex since it includes not only bacteria but also ciliated protozoans, parasites, and some symbiotic nematodes (120).

In another study, the bacterial flora of hibernating and non-hibernating leopard frogs (*R. pipiens*) was studied by classic microbiological cultivation methods. These adult frogs host 10^10^ and 10^9^ bacteria per gram of intestinal contents and mucosal scraping, respectively. The intestinal flora was reported to be similar to that of mammals and birds, with *Bacteroides* as dominant organisms. Strictly anaerobic bacteria were also isolated, including butyrogenic and acetogenic bacteria. The largest microorganisms inhabiting leopard frog’s gut are protozoans and several ciliates have been identified such as species of *Opalina*, *Nyctotherus*, and *Balantidium*.

**Figure 2:**
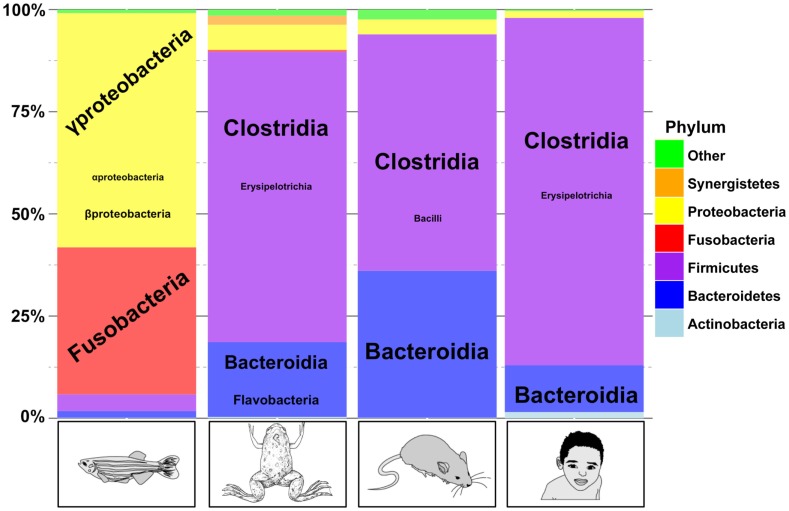
**A comparison of gut microbiome taxonomic profiles in fish, frog, mice, and human**. Comparison between four adult vertebrate gut microbiomes. Phylum are represented by colors, and class for the most abundant phylum are represented according to the font size. This figure was adapted from Kostic et al. (121) and from available data on SRA database for *H. sapiens* (ERR139249) (122), *M. musculus* (SRR513991) (123), *D. rerio* (ERR012013) (124), and *X. laevis* (SRX247015) (57). Data were submitted to the Ribosomal Database Project Classifier tool for taxonomy classification (125). This graph was generated using R (http://cran.r-project.org/bin/).

More recently, the microbial diversity in adult *X. laevis* gut was analyzed using 16S ribosomal RNA sequencing (57). The microbial composition was studied in distinct portions of the gastrointestinal tract including stomach, small intestine, and large intestine. As in human and mice, Firmicutes, Bacteroidetes, and Proteobacterias are the major component of the frog gut microbiome (Figure 2). A few Flavobacteria were also observed. Flavobacteria are also observed in zebrafish gut microbiome but they are absent from mammalian gut microbiomes. A significant variation in the microbiome composition was observed from one animal to another but also from one part of the intestine system to another one. The authors studied the impact of T-cell deficiency resulting from thymectomy at early developmental stage on the gut microbial composition, and they did not observe significant variation of the gut microbiome as a consequence of thymectomy.

We know that most amphibians experience a dramatic remodeling of the intestine during metamorphosis accompanied by a change in feeding behavior. Yet, the evolution of the amphibian gut microbiome during metamorphosis is still relatively unknown. Metamorphosis is associated with a structural modification of the gut, a change in the gut pH, and a switch of diet habit from herbivorous tadpoles to omnivorous adults. Thus, the gut microbiota composition is expected to change between tadpoles and adults. In one study, the relationships between the gut microflora and metamorphosis were investigated in two anuran species, *Bufo terrestris* and *Pseudacris crucifer* (116). Gram-negative bacteria were more abundant in froglets than in tadpoles in both species. *Aeromonas hydrophila* was found in both tadpoles and froglets, and in increased numbers in *P. crucifer* froglets in comparison to tadpoles. These increased levels of bacteria at a developmental stage associated with immunosuppression may increase the susceptibility to bacterial infections during metamorphosis. In another study, Kohl et al. described the evolution of the gut microbiota composition during metamorphosis in the northern leopard frogs (*Lithobates pipiens*) by 16S rRNA sequencing (126). The authors sampled the whole digesta of laboratory-bred animals. This study revealed that tadpoles and adult frogs had a microbiota dominated by Proteobacteria and Firmicutes. Phylum and genus analysis between tadpoles and frogs let them to conclude that tadpoles gut microbiota composition is similar to invertebrate or fish, while adult frogs gut microbiota resembles that of amniotes (mammals, birds, reptiles…). They observed not only a change in the composition of the microbiota but also a diminution of the microbial diversity after metamorphosis. They explained this modification of the gut microbiota composition by the changes of diet, and the evolution of the gastrointestinal system during metamorphosis.

We can conclude from these two recent studies that the frog gut microbiome composition resembles that of terrestrial mammals and is different from the fish (57, 126). The tadpole gut microbiome situation seems to be intermediate. One question still unanswered is when does the microbiome colonize tadpole’s gut and other mucosa.

## Perspectives

The function of the vertebrate intestinal immune system depends on the fine balance between effector and regulatory mechanisms. This balance is important not only for preventing diseases, but also for providing flexibility and thus instructing the appropriate immune response. Dysfunctions in any component of this homeostasis can cause inflammatory diseases of the gastrointestinal tract.

Since amphibian and other non-mammalian vertebrates lack some gut secondary lymphoid structures in comparison to mammals, where does the balance of the gut mucosal immunity occur? It is now well established that the programed development of lymph nodes and PPs during mammalian ontogeny requires LTi cells (see Background on the Gut Mucosal Immune System), which express the nuclear hormone receptor RORgt. It is also known that after birth, gut LTi cells cluster into cryptopatches, the precursor structures of ILFs, which are induced by symbiotic bacteria and are involved in the maintenance of intestinal homeostasis. On the other hand, it has been demonstrated that tertiary lymphoid tissues also form in the postnatal period in response to antigenic stimuli (127). As described above (see The Thymus), ILFs and tertiary lymphoid tissues may contribute to the local protective immune response. Thus, the generation of artificial tertiary lymphoid tissues has even been proposed as a novel immunotherapy to induce effective anti-tumor immune responses (31).

**Figure 3:**
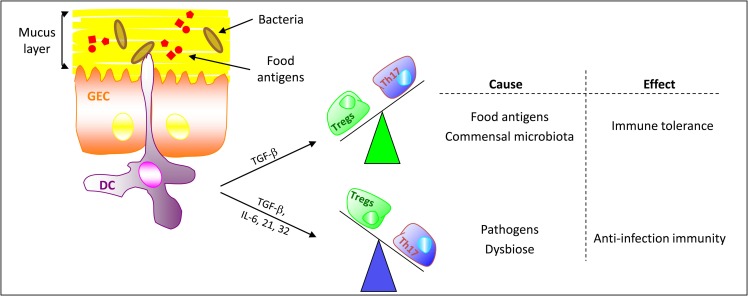
**Microbiota and food antigens regulate the gut immune balance**. In normal, non-pathological, conditions tolerogenic DCs promote the differentiation of naive CD4^+^ T cells toward Tregs *via* the expression of transforming growth factor-β (TGF-β). Thus, TGF-β and Tregs are critical in maintaining self-tolerance and immune homeostasis. In the presence of danger signals (pathogens, dysbiosis, …), activated DCs co-stimulate naive CD4^+^ T cells to differentiate into effector Th17 cells. (GEC, gut epithelial cell)

Based on these outcomes, one could expect that the amphibian microbiota would influence the formation of lymphoid structures. Such structures in amphibian’s gut mucosa would assume the functions of MLNs and PPs. This hypothesis could explain the defense mechanisms developed in the gut mucosa of amphibians and other non-mammalian vertebrates, as well as the microbiota involvement in the regulation of the gut immune balance (Figure 3). Indeed, key features of host–microbe relationships are the ability of the gut microbiota to modify dietary nutrient metabolism and to modulate the immune balance in all vertebrates. For example, the zebrafish gut microbiota influences some aspects of the lipid metabolism (96).

Yet, *Xenopus* are closer relatives to mammals than the teleost zebrafish. This is true for both genetic and physiological traits and thus the frog models represent an attractive model (5, 128, 129). As we reviewed here, it appears that the intestinal microbiome of *Xenopus* adults is characterized by the abundance of Bacteroidetes, Firmicutes, and Actinobacteria and resembles in this to the mammalian intestinal microbiome (Figure 2). Similarly, the adult amphibian skin microbiome plays an important immunological role. We are performing taxonomic and metabolic profiles of *X. tropicalis* microbiome to obtain a more detailed picture of the gut microflora and of its activity. Once these profiles will be obtained, we will be able to explore the interactions between the gut microbiome and the mucosal immune system in the *Xenopus* model. Indeed, recent reports demonstrate that modifications of the gut microbial composition modulate the immune responses and can reduce inflammation (130).

It is now well established that changes in environmental factors such as the dietary regimens can alter microbiota composition. This external dietary intervention could decrease the susceptibility to inflammatory diseases by modulating gut immunity and could restore a healthy gut microbiota after an infection. An easily and convenient strategy is represented by the therapeutic use of live microorganisms such as probiotics, microbe-derived metabolites, or prebiotics. Probiotic or prebiotic can be used for prophylactic or therapeutic interventions either for gut inflammation or extra-intestinal diseases associated to the immune system dysregulation (131). The engineering of probiotics is a promising line of research for producing specific therapeutic effects or producing drugs according to the patient physiological status (132).

In conclusion, we recapitulated the latest literature showing how the immune system of amphibians resembles that of mammals, in terms of molecules, cells, tissues, and functions. Furthermore, we highlighted the studies showing that amphibians harbor an abundant microflora. In particular, this microflora colonizes mucosal tissues and is abundant in the amphibian gastrointestinal tract as well as in the skin. Although several authors have described that a microflora is abundant at the onset of tadpole stages, this knowledge is descriptive and derived from studies of natural populations and in non-model amphibian species. Future research on the *Xenopus* small-animal model is warranted to study host–microbiota interactions in relation to the mucosal immune system. *Xenopus* provides low-cost housing and a very rapid development into tadpoles that feed on microorganisms plus other advantages such as transparency small size, availability in large numbers, genetic, and physiologic resemblance. All this makes *Xenopus* closer to mammals than fish (128, 133). Thus, *Xenopus* tadpoles can become an attractive alternative tool enabling the integrated study of host–microbiota interactions.

## Conflict of Interest Statement

The authors declare that the research was conducted in the absence of any commercial or financial relationships that could be construed as a potential conflict of interest.

## Abbreviations

AIRE, autoimmune regulator; CTX, thymocyte-specific antigen of *Xenopus*; DAMP, damage-associated molecular pattern; DC, dendritic cell; DETCs, dendritic epidermal T cells; dpf, days post-fertilization; IBD, inflammatory bowel disease; ILC, innate lymphoid cell; ILF, isolated lymphoid follicle; LTi, lymphoid-tissue inducer; MAIT, mucosal-associated invariant T cells; MAMP, microbial-associated molecular pattern; MHC, major histocompatibility complex; MLN, mesenteric lymph node; NK, natural killer cells; PP, Peyer’s patch; PRR, pattern recognition receptors; RA, retinoic acid; TEC, thymic epithelial cell; Treg, regulatory T cell.
